# Supplementary material for: Effect of proprioceptive neuromuscular facilitation on patients with chronic ankle instability: A systematic review and meta-analysis
Source: PLoS One. 2025 Jan 9;20(1):e0311355. doi: 10.1371/journal.pone.0311355 (PMC11717224; doi:10.1371/journal.pone.0311355)
Supplement: S1 File — (DOCX) [file pone.0311355.s001.docx]

**Supplementary Table 1. Search strategy for each database**

| Search engine | Search query | Date of search |
| --- | --- | --- |
| CNKI/VIP/ Wanfang Data /CBM | #1 主题 (Topic) =慢性踝关节不稳(chronic ankle instability、CAI) OR踝关节不稳(ankle instability) OR功能性踝关节不稳(function ankle instability、FAI) OR踝关节不稳(ankle instability) OR踝关节扭伤(ankle sprain)  #2 主题 (Topic) =本体感觉神经肌肉促进技术/本体感觉神经肌肉促进法(proprioceptive neuromuscular facilitation/ Proprioceptive Neuromuscular Facilitation Techniques/PNF) OR交互抑制(reciprocal inhibition) OR螺旋对角线(Spiral diagonal)  #3 #1 AND #2 | April 10, 2024 |
| Pubmed | #1 ankle sprain [MeSH Major Topic]  #2 chronic ankle instability [Text Word]  #3 CAI [Text Word]  #4 function ankle instability [Text Word]  #5 FAI [Text Word]  #6 ankle instability [Text Word]  #7 ankle injury [Text Word]  #8 #1 OR #2 OR #3 OR #4 OR #5 OR #6 OR #7  #9 muscle stretching exercises [MeSH Terms]  #10 proprioceptive neuromuscular facilitation [Text Word]  #11 Proprioceptive Neuromuscular Facilitation Techniques [Text Word]  #12 PNF [Text Word]  #13 reciprocal inhibition [Text Word]  #14 Spiral diagonal [Text Word]  #15 PNF stretching [Text Word]  #16 #9 OR #10 OR #11 OR #12 OR #13 OR #14 OR #15  #17 #8 AND #16 | April 10, 2024 |
| Web of science | #1 TS = (‘ankle sprain’ OR ‘chronic ankle instability’ OR ‘CAI’ OR ‘function ankle instability’ OR ‘FAI’ OR ‘ankle instability’ OR ‘ankle injury’)  #2 TS = (‘muscle stretching exercises’ OR ‘proprioceptive neuromuscular facilitation’ OR ‘Proprioceptive Neuromuscular Facilitation Techniques’ OR ‘PNF’ OR ‘reciprocal inhibition’ OR ‘Spiral diagonal’ OR ‘PNF stretching’)  #3 #1 AND #2  Databases = SCI-EXPANDED, SSCI, A&HCI, CPCI-S, CPCI-SSH, ESCI | April 10, 2024 |
| EBSCO(Medline, CINAHL, SPORT Discus, Rehabilitation & Sports Medicine Source), | S1 ‘ankle sprain’ OR ‘chronic ankle instability’ OR ‘CAI’ OR ‘function ankle instability’ OR ‘FAI’ OR ‘ankle instability’ OR ‘ankle injury’  S2 ‘muscle stretching exercises’ OR ‘proprioceptive neuromuscular facilitation’ OR ‘Proprioceptive Neuromuscular Facilitation Techniques’ OR ‘PNF’ OR ‘reciprocal inhibition’ OR ‘Spiral diagonal’ OR ‘PNF stretching’  #3 #1 AND #2 | April 10, 2024 |
| Embase | #1‘ankle sprain’ OR ‘chronic ankle instability’ OR ‘CAI’ OR ‘function ankle instability’ OR ‘FAI’ OR ‘ankle instability’ OR ‘ankle injury’  #2 ‘muscle stretching exercises’ OR ‘proprioceptive neuromuscular facilitation’ OR ‘Proprioceptive Neuromuscular Facilitation Techniques’ OR ‘PNF’ OR ‘reciprocal inhibition’ OR ‘Spiral diagonal’ OR ‘PNF stretching’  #3 #1 AND #2 | April 10, 2024 |
| Cochrane Library | S1 dance OR dance training OR dancing exercises OR aerobic dance  S2 muscle stretching exercises OR proprioceptive neuromuscular facilitation OR Proprioceptive Neuromuscular Facilitation Techniques OR PNF OR reciprocal inhibition OR Spiral diagonal OR PNF stretching  S3 S1 AND S2 | April 10, 2024 |
| Science Direct | S1 dance OR dance training OR dancing exercises OR aerobic dance  S2 muscle stretching exercises OR proprioceptive neuromuscular facilitation OR Proprioceptive Neuromuscular Facilitation Techniques OR PNF OR reciprocal inhibition OR Spiral diagonal OR PNF stretching  S3 S1 AND S2 | April 10, 2024 |
| ProQuest | S1 dance OR dance training OR dancing exercises OR aerobic dance  S2 muscle stretching exercises OR proprioceptive neuromuscular facilitation OR Proprioceptive Neuromuscular Facilitation Techniques OR PNF OR reciprocal inhibition OR Spiral diagonal OR PNF stretching  S3 S1 AND S2 | April 10, 2024 |

Table note: CNKI: China National Knowledge Infrastructure; VIP:VIP database; CBM: China Biology Medicine disc
