# Supplementary material for: Effect of proprioceptive neuromuscular facilitation on patients with chronic ankle instability: A systematic review and meta-analysis
Source: PLoS One. 2025 Jan 9;20(1):e0311355. doi: 10.1371/journal.pone.0311355 (PMC11717224; doi:10.1371/journal.pone.0311355)
Supplement: S2 File — (DOCX) [file pone.0311355.s002.docx]

Name of data extractors: Yikun Yin, Yinghang Luo, and Yongsheng Liu.

Date of data extraction: May 31, 2024

1. Cumberland Ankle Instability Tool (CAIT)

| study | year | case_n | case_mean | case_SD | control_n | contral_mean | contral_SD |
| --- | --- | --- | --- | --- | --- | --- | --- |
| RENYI | 2023 | 24 | 102.28 | 18.92 | 24 | 92.78 | 16.96 |
| JIESONG Y | 2023 | 17 | 36.82 | 1.51 | 17 | 33.06 | 1.82 |
| LINHUA X | 2021 | 25 | 23.14 | 4.11 | 25 | 20.13 | 2.16 |
| MENGFAN S | 2017 | 10 | 23.1 | 4.7 | 10 | 20 | 2.21 |
| Alahmari K A | 2020 | 20 | 95.3 | 3.4 | 20 | 95.5 | 3.7 |
| XUEPENG X | 2020 | 29 | 23.48 | 2.83 | 29 | 20.09 | 2.83 |
| PANG Min | 2019 | 30 | 20.41 | 2.63 | 30 | 17.47 | 2.42 |

2. Y Balance Test (YBT)

| study | year | case_n | case_mean | case_SD | control_n | contral_mean | contral_SD |
| --- | --- | --- | --- | --- | --- | --- | --- |
| RENYI | 2023 | 24 | 102.28 | 18.92 | 24 | 92.78 | 16.96 |
| YUDUO L | 2022 | 17 | 36.82 | 1.51 | 17 | 33.06 | 1.82 |
| Hall E A | 2015 | 25 | 23.14 | 4.11 | 25 | 20.13 | 2.16 |

3. Star Excursion Balance Test (SEBT)

| study | year | case_n | case_mean | case_SD | control_n | contral_mean | contral_SD | group |
| --- | --- | --- | --- | --- | --- | --- | --- | --- |
| JIESONG Y | 2023 | 17 | 85.82 | 1.51 | 17 | 76.41 | 1.69 | SEBT-ANT |
| LINHUA X | 2021 | 25 | 81.03 | 3.61 | 25 | 78.81 | 3.23 | SEBT-ANT |
| MENGFAN S | 2017 | 10 | 81.05 | 3.63 | 10 | 77.3 | 5.02 | SEBT-ANT |
| Alahmari K A | 2020 | 20 | 70.2 | 4.1 | 20 | 70.7 | 7 | SEBT-ANT |
| JIESONG Y | 2023 | 17 | 85.82 | 1.51 | 17 | 76.41 | 1.69 | SEBT-ALAT |
| LINHUA X | 2021 | 25 | 71.81 | 4.2 | 25 | 69.34 | 3.26 | SEBT-ALAT |
| MENGFAN S | 2017 | 10 | 70.83 | 4.22 | 10 | 67.37 | 3.28 | SEBT-ALAT |
| Alahmari K A | 2020 | 20 | 76.4 | 3.8 | 20 | 76.1 | 4 | SEBT-ALAT |
| JIESONG Y | 2023 | 17 | 89.94 | 2.11 | 17 | 82.94 | 3.6 | SEBT-AMED |
| LINHUA X | 2021 | 25 | 89.44 | 6.81 | 25 | 84.21 | 5.27 | SEBT-AMED |
| MENGFAN S | 2017 | 10 | 89.47 | 6.84 | 10 | 83.24 | 5.28 | SEBT-AMED |
| Alahmari K A | 2020 | 20 | 84.3 | 5.7 | 20 | 84.1 | 5.8 | SEBT-AMED |
| JIESONG Y | 2023 | 17 | 100.29 | 2.31 | 17 | 85.94 | 3.4 | SEBT-MED |
| LINHUA X | 2021 | 25 | 93.01 | 3.86 | 25 | 87.79 | 8.02 | SEBT-MED |
| MENGFAN S | 2017 | 10 | 93.02 | 3.89 | 10 | 86.54 | 8.06 | SEBT-MED |
| Alahmari K A | 2020 | 20 | 98.3 | 3.7 | 20 | 96.8 | 3.3 | SEBT-MED |
| JIESONG Y | 2023 | 17 | 102.65 | 1.54 | 17 | 89.29 | 2.67 | SEBT-POST |
| LINHUA X | 2021 | 25 | 103.69 | 8.36 | 25 | 99.42 | 5.43 | SEBT-POST |
| MENGFAN S | 2017 | 10 | 102.24 | 8.35 | 10 | 95.32 | 5.45 | SEBT-POST |
| Alahmari K A | 2020 | 20 | 92.4 | 4.5 | 20 | 90.9 | 5.3 | SEBT-POST |
| JIESONG Y | 2023 | 17 | 82.35 | 1.73 | 17 | 69.18 | 1.51 | SEBT-LAT |
| LINHUA X | 2021 | 25 | 87.82 | 6.34 | 25 | 84.32 | 5.23 | SEBT-LAT |
| MENGFAN S | 2017 | 10 | 85.4 | 6.39 | 10 | 81.89 | 6.63 | SEBT-LAT |
| Alahmari K A | 2020 | 20 | 93.9 | 4.1 | 20 | 91.8 | 5.1 | SEBT-LAT |
| JIESONG Y | 2023 | 17 | 94.41 | 1.77 | 17 | 83.35 | 2.34 | SEBT-PLAT |
| LINHUA X | 2021 | 25 | 102.32 | 9.65 | 25 | 96.58 | 5.03 | SEBT-PLAT |
| MENGFAN S | 2017 | 10 | 99.42 | 9.7 | 10 | 90.85 | 8.21 | SEBT-PLAT |
| Alahmari K A | 2020 | 20 | 96.7 | 3.2 | 20 | 95.1 | 2.9 | SEBT-PLAT |
| JIESONG Y | 2023 | 17 | 104.06 | 1.64 | 17 | 94.41 | 3.37 | SEBT-PMED |
| LINHUA X | 2021 | 25 | 103.26 | 7.02 | 25 | 98.28 | 7.13 | SEBT-PMED |
| MENGFAN S | 2017 | 10 | 103.23 | 7.16 | 10 | 96.29 | 7.28 | SEBT-PMED |
| Alahmari K A | 2020 | 20 | 98.3 | 3.2 | 20 | 97.2 | 3 | SEBT-PMED |

4. Muscle Strength

| study | year | case_n | case_mean | case_SD | control_n | contral_mean | contral_SD | group |
| --- | --- | --- | --- | --- | --- | --- | --- | --- |
| RENYI | 2023 | 24 | 1.13 | 0.41 | 24 | 0.83 | 0.45 | Plantar flexion |
| LINHUA X | 2021 | 25 | 43.96 | 6.63 | 25 | 41.56 | 6.54 | Plantar flexion |
| MENGFAN S | 2017 | 10 | 41.51 | 18.72 | 10 | 46.47 | 6.64 | Plantar flexion |
| Hall E A | 2015 | 13 | 259.3 | 50.8 | 13 | 255.4 | 67 | Plantar flexion |
| Alahmari K A | 2020 | 20 | 15.1 | 0.9 | 20 | 14.3 | 1.5 | Plantar flexion |
| RENYI | 2023 | 24 | 0.43 | 0.24 | 24 | 0.31 | 0.17 | Dorsal flexion |
| LINHUA X | 2021 | 25 | 28.34 | 6.29 | 25 | 27.87 | 6.73 | Dorsal flexion |
| MENGFAN S | 2017 | 10 | 23.36 | 11.56 | 10 | 27.99 | 6.87 | Dorsal flexion |
| Hall E A | 2015 | 13 | 299.7 | 34 | 13 | 267.2 | 50.4 | Dorsal flexion |
| Alahmari K A | 2020 | 20 | 11.7 | 0.7 | 20 | 11.3 | 0.8 | Dorsal flexion |
| LINHUA X | 2021 | 25 | 21.89 | 6.91 | 25 | 22.87 | 4.66 | Varus |
| MENGFAN S | 2017 | 10 | 20.93 | 9.94 | 10 | 22.91 | 4.67 | Varus |
| Hall E A | 2015 | 13 | 212.6 | 44.4 | 13 | 188.2 | 47.2 | Varus |
| LINHUA X | 2021 | 25 | 16.39 | 6.97 | 25 | 18.97 | 6.08 | Eversion |
| MENGFAN S | 2017 | 10 | 16.27 | 6.08 | 10 | 18.99 | 6.09 | Eversion |
| Hall E A | 2015 | 13 | 183.9 | 27.9 | 13 | 176 | 43 | Eversion |

5. VAS

| study | year | case_n | case_mean | case_SD | control_n | contral_mean | contral_SD |
| --- | --- | --- | --- | --- | --- | --- | --- |
| RENYI | 2023 | 24 | 2.06 | 1.52 | 24 | 2.93 | 1.84 |
| Hall E A | 2015 | 13 | 2.8 | 0.9 | 13 | 4.9 | 2.2 |
| Lazarou L | 2017 | 11 | 0.4 | 0.4 | 11 | 1.1 | 1.3 |
| Alahmari K A | 2020 | 20 | 1.6 | 0.8 | 20 | 2 | 0.8 |
