# Supplementary material for: Effect of proprioceptive neuromuscular facilitation on patients with chronic ankle instability: A systematic review and meta-analysis
Source: PLoS One. 2025 Jan 9;20(1):e0311355. doi: 10.1371/journal.pone.0311355 (PMC11717224; doi:10.1371/journal.pone.0311355)
Supplement: S3 File — (DOCX) [file pone.0311355.s003.docx]

**Unmatched study design(n-11)**

1.Rees S, Murphy A, Watsford M, McLachlan K. The effect of proprioceptive neuromuscular facilitation stretching on stiffness of the ankle. (Abstract). Journal of Science & Medicine in Sport. 2005;8(4 Supplement):21-. PubMed PMID: SPHS-1012769.

2.Haff GG. Roundtable Discussion: Flexibility Training. Strength & Conditioning Journal. 2006;28(2):64-85. doi: 10.1519/1533-4295(2006)028[0064:RDFT]2.0.CO;2. PubMed PMID: 20567584.

3.Ivins D. Acute ankle sprain: an update. American family physician. 2006;74(10):1714-20. PubMed PMID: 17137000.

4.Nicola TL, El Shami A. Rehabilitation of Running Injuries. Clinics in Sports Medicine. 2012;31(2):351-72. doi: 10.1016/j.csm.2011.10.002.

5.Espí-López GV, López-Martínez S, Inglés M, Serra-Añó P, Aguilar-Rodríguez M. Effect of manual therapy versus proprioceptive neuromuscular facilitation in dynamic balance, mobility and flexibility in field hockey players. A randomized controlled trial. Physical Therapy in Sport. 2018;32:173-9.

5.Sharma A, Saxena A. Combined Effect of Pelvic Proprioceptive Neuromuscular Facilitation with Core Strengthening on Trunk Control, Balance, and Gait in Paraplegia. International journal of applied & basic medical research. 2023;13(4):263-5. doi: 10.4103/ijabmr.ijabmr_190_23. PubMed PMID: 38229730.

6.Fanji Q, Jinfeng L. Effects of different modes of proprioceptive neuromuscular facilitation interventions on balance ability of functional ankle instability. Chinese Journal of Rehabilitation Medicine. 2022;37(6).

7.Jayaseelan VK, Senthil P, Rathnamala D, Dolla K. A study to compare the effectiveness of Kinesio taping versus contract relax proprioceptive neuromuscular facilitation stretching of plantar flexors flexibility in amateur badminton players. Drug Invention Today. 2020;14(1):27-30.

8.Zeng X, Zhu G, Zhang M, Xie SQ. Reviewing Clinical Effectiveness of Active Training Strategies of Platform-Based Ankle Rehabilitation Robots. Journal of healthcare engineering. 2018;2018:2858294. doi: 10.1155/2018/2858294. PubMed PMID: 29675142.

9.Kay A, Dods S, Blazevich A, Kay AD, Blazevich AJ. Acute effects of contract-relax (CR) stretch versus a modified CR technique. European Journal of Applied Physiology. 2016;116(3):611-21. doi: 10.1007/s00421-015-3320-8. PubMed PMID: 112926710.

10.Hall EA, Frank J, Docherty CL. The Effectiveness of Strength Training Protocols on Strength Development in Participants With Chronic Ankle Instability: A Critically Appraised Topic. International Journal of Athletic Therapy & Training. 2015;20(1):13-7. doi: 10.1123/ijatt.2014-0049. PubMed PMID: 100166648.

11.Rees SS, Murphy AJ, Watsford ML, McLachlan KA, Coutts AJ. EFFECTS OF PROPRIOCEPTIVE NEUROMUSCULAR FACILITATION STRETCHING ON STIFFNESS AND FORCE‐PRODUCING CHARACTERISTICS OF THE ANKLE IN ACTIVE WOMEN. Journal of Strength and Conditioning Research. 2007;21:572–7.

**Irrelevant outcome indicators(n-8)**

1.Masatoshi N, Tome l, Takahiro T, Noriaki I. Acute Effects of Stretching on Passive Properties of Human Gastrocnemius Muscle-Tendon Unit: Analysis of Differences Between Hold-Relax and Static Stretching. Journal of Sport Rehabilitation. 2015;24(3):286-92. doi: 10.1123/jsr.2014-0164. PubMed PMID: 108890501.

2.Lazarou L, Kofotolis N, Malliou P, Kellis E. Effects of two proprioceptive training programs on joint position sense, strength, activation and recurrent injuries after ankle sprains. Isokinetics & Exercise Science. 2017;25(4):289-300. doi: 10.3233/IES-171146. PubMed PMID: 126799973. Language: English. Entry Date: 20171219. Revision Date: 20181203. Publication Type: Journal Article.

3.Chen H, Hu W, Liu Y, Na J, Li Q, Wan X. The impact of whole-body vibration training and proprioceptive neuromuscular facilitation on biomechanical characteristics of lower extremity during cutting movement in individuals with functional ankle instability: A parallel-group study. Clinical biomechanics (Bristol, Avon). 2024;113:106208. doi: 10.1016/j.clinbiomech.2024.106208. PubMed PMID: 38377653.

4.Choi JH. Effects of elastic band exercise using proprioceptive neuromuscular facilitation on strength and dynamic balance of adults with ankle instability. Medico-Legal Update. 2019;19(2):628-34. doi: 10.5958/0974-1283.2019.00246.9.

6.Boob M, Phansopkar P. Effect of foot core exercises vs ankle proprioceptive neuromuscular facilitation on pain, range of motion, and dynamic balance in individuals with plantar fasciitis: a comparative study. F1000Research. 2024;12:765. doi: 10.12688/f1000research.136828.2

7.Wu, Kevin. The Effects of Proprioceptive Neuromuscular Facilitation Techniques with Core Strengthening on Dynamic Balance in Men with Chronic Ankle Instability. Azusa Pacific University ProQuest Dissertations & Theses.2019.

8.Al-Khafaji WSM, Al-Masoodi FHK. The Effect of Stretching Exercises by Proprioceptive Neuromuscular Facilitation (Pnf) Of Sensory Receptors in the Rehabilitation of Ankle Injury for Athletes. Revista iberoamericana de psicología del ejercicio y el deporte. 2023;18(4):355-60.

**Failed to extract outcome data(n-1)**

1.Choi J-H. Effects of Elastic Band Exercise Using Proprioceptive Neuromuscular Facilitation on Strength and Dynamic Balance of Adults with Ankle Instability. Medico Legal Update. 2019;19:628-34. doi: 10.37506/mlu.v19i2.848.
