# Supplementary material for: Effect of proprioceptive neuromuscular facilitation on patients with chronic ankle instability: A systematic review and meta-analysis
Source: PLoS One. 2025 Jan 9;20(1):e0311355. doi: 10.1371/journal.pone.0311355 (PMC11717224; doi:10.1371/journal.pone.0311355)
Supplement: S5 File — (DOCX) [file pone.0311355.s005.docx]

**Supplementary Figure 1 Meta-analysis of the effect of PNF on Star Excursion Balance Test (Exclusion heterogeneity research)**

**
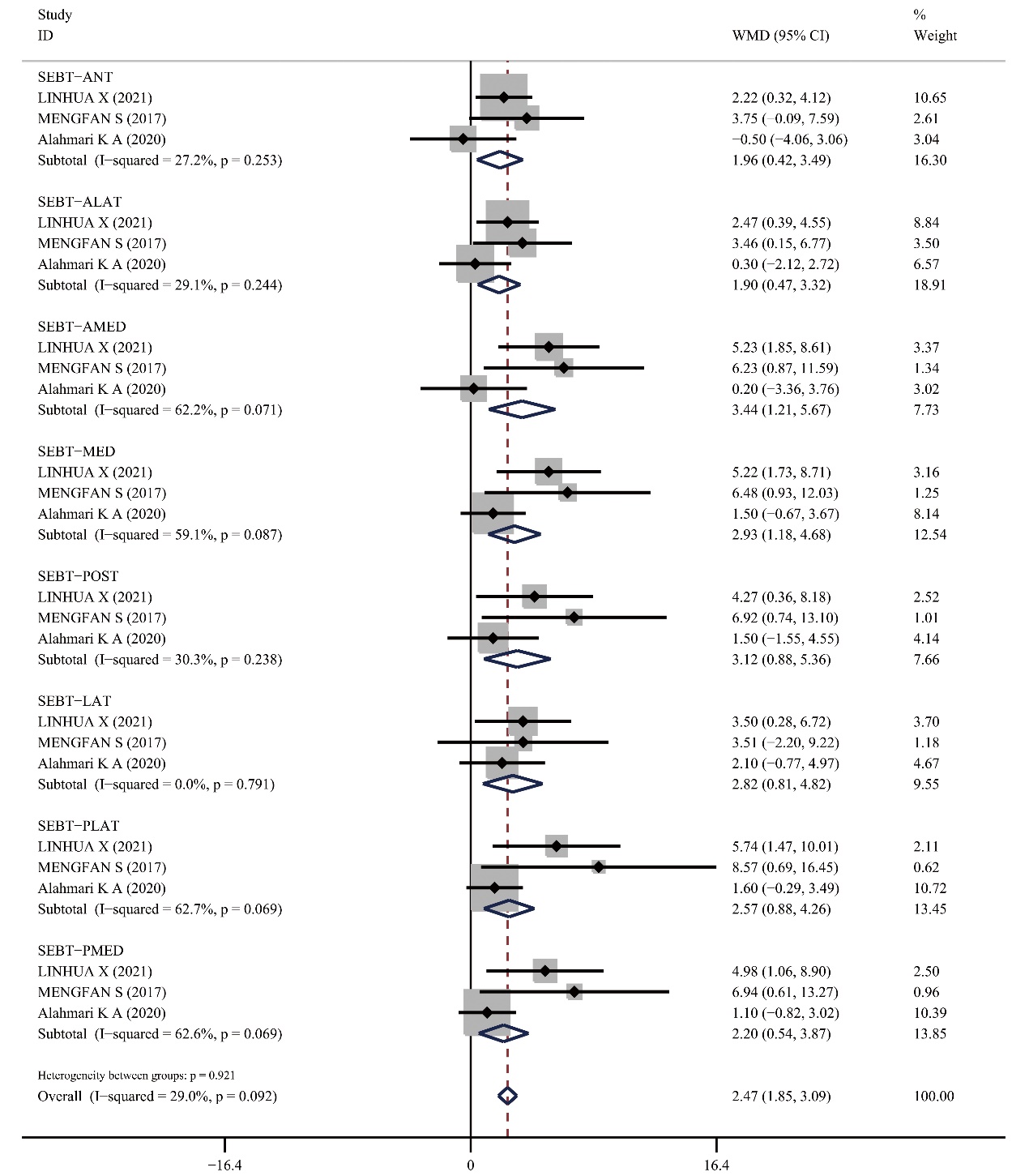
**

**Supplementary Figure 2 Meta-analysis of the effect of PNF on Ankle instability questionnaire (Exclusion heterogeneity research)**

**
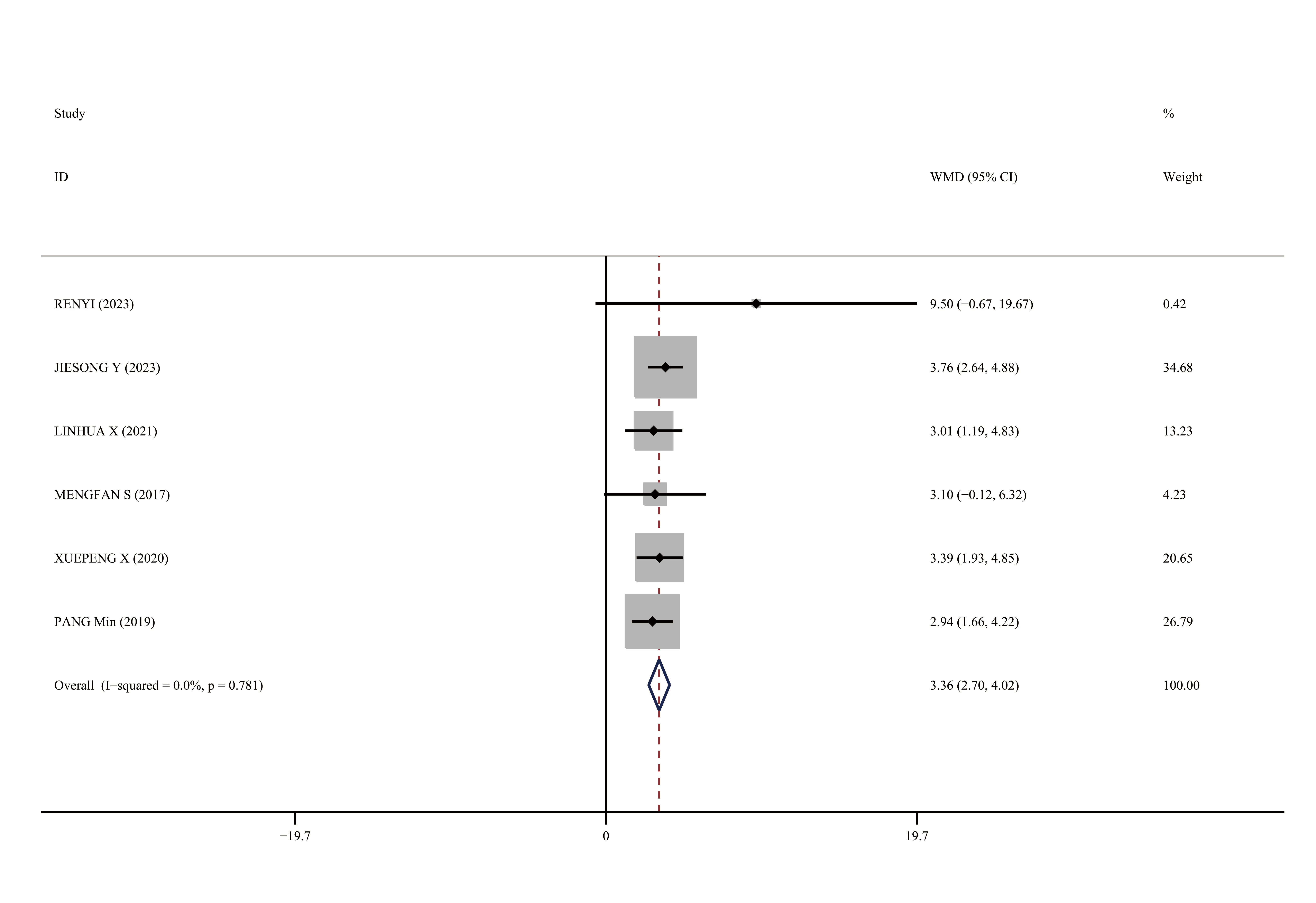
**
