# Supplementary material for: Effect of proprioceptive neuromuscular facilitation on patients with chronic ankle instability: A systematic review and meta-analysis
Source: PLoS One. 2025 Jan 9;20(1):e0311355. doi: 10.1371/journal.pone.0311355 (PMC11717224; doi:10.1371/journal.pone.0311355)

**Supplementary Figure 3 Ankle instability questionnaire publication bias graph of the included literature**

**
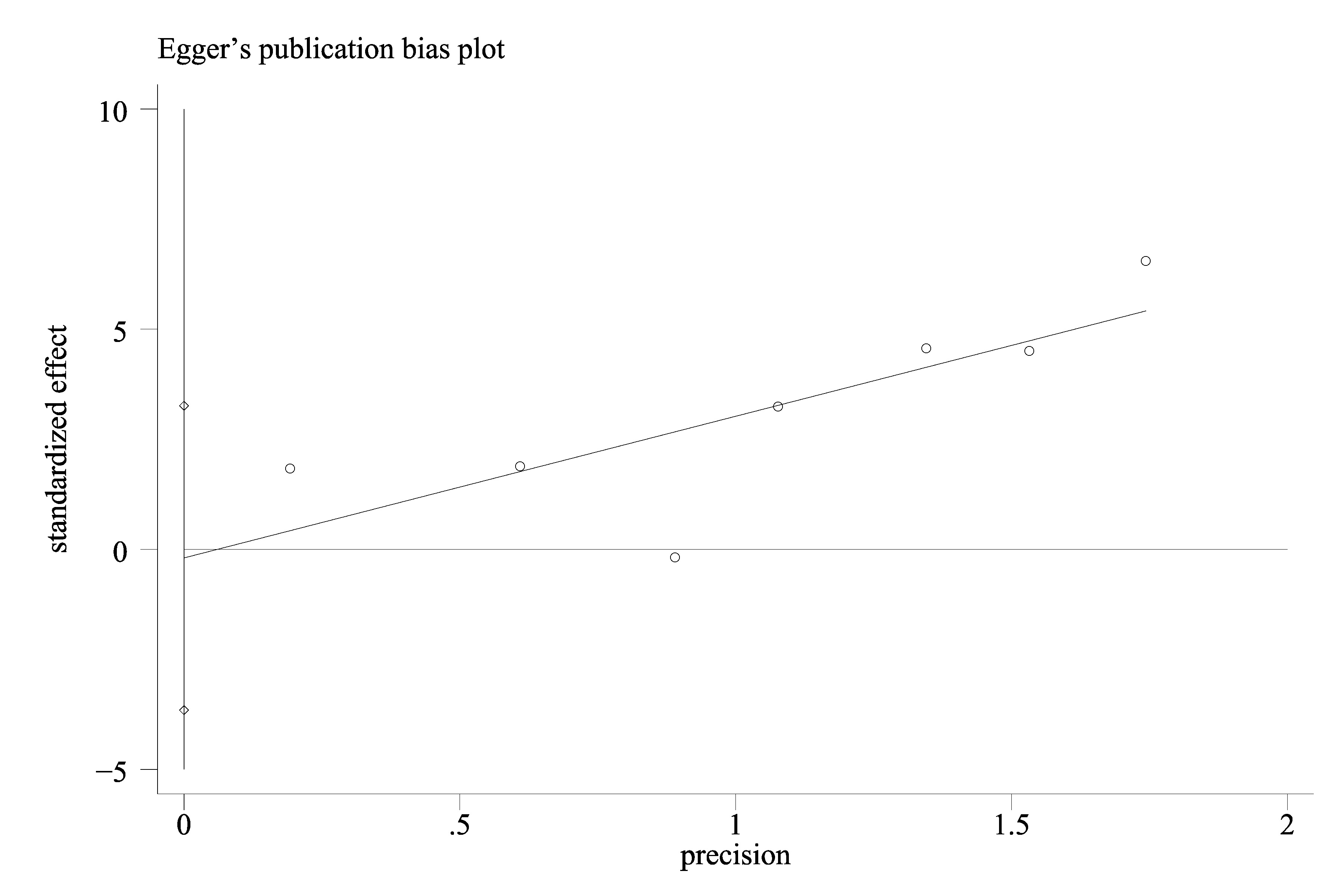
**

**Supplementary Figure 4 Plantar flexion publication bias graph of the included literature**


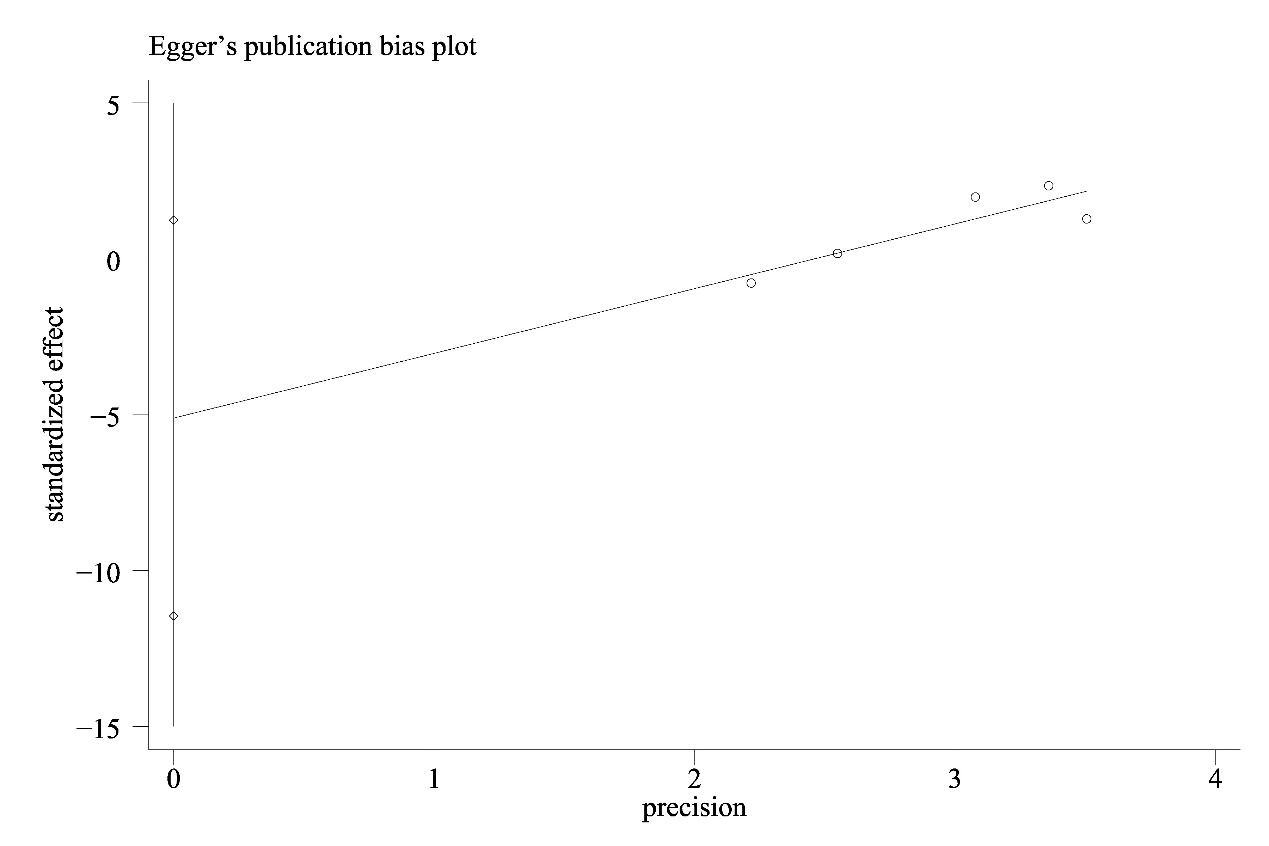


**Supplementary Figure 5 Dorsal flexion publication bias graph of the included literature**


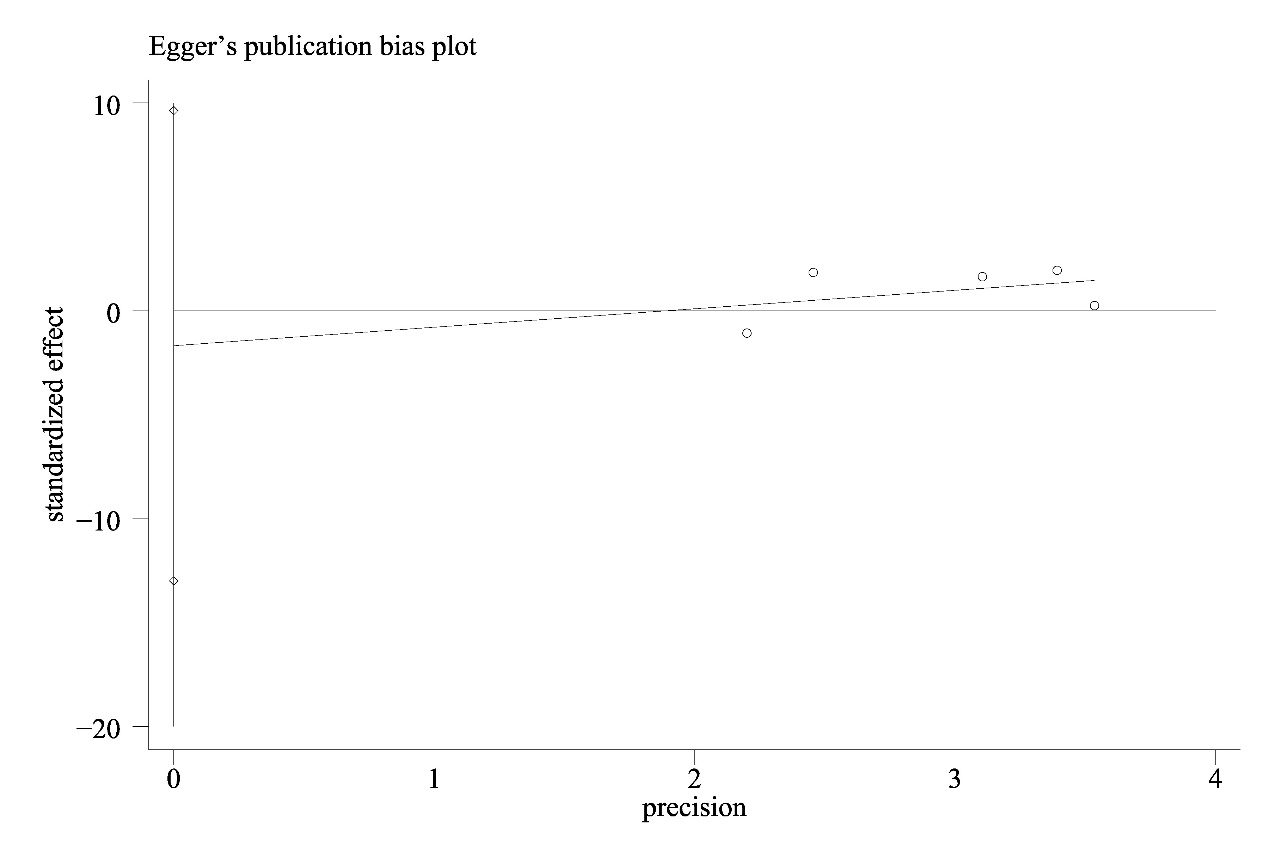

Supplement: S6 File — (DOCX) [file pone.0311355.s006.docx]
