# Supplementary material for: Effect of proprioceptive neuromuscular facilitation on patients with chronic ankle instability: A systematic review and meta-analysis
Source: PLoS One. 2025 Jan 9;20(1):e0311355. doi: 10.1371/journal.pone.0311355 (PMC11717224; doi:10.1371/journal.pone.0311355)
Supplement: Attachment — Submitted filename: Response to Reviewers.docx [file pone.0311355.s007.docx]

Dear Reviewer

We deeply appreciate your valuable comments concerning our manuscript. The suggestions are all very helpful for revision and improvement. We have carefully studied these comments and made related modifications, which we wish could meet with your approval.

The modified content has been marked yellow in the text.

**1. Include a detailed flowchart of the study selection process in the methods section.**

**Thank you for this suggestion. Revisions have been made to the manuscript.**

2.2 Literature Inclusion、Exclusion Criteria and Outcome Indicator

According to the PICOS principle, the inclusion、exclusion criteria and outcome indicator for the literature are as follows (See Table 1):

Table 1. PICOS framework

| Parameter | Inclusion criteria | Exclusion criteria |
| --- | --- | --- |
| P (population) | Adults (>18yrs) population from chronic ankle instability：  1) Subjects had at least one history of ankle sprain in the past 12 months, causing pain and swelling, and the time to lose normal function within 1 day or more  2) The affected ankle of subjects felt "soft leg", and/or repeatedly sprains and/or "un-stable" | Non-chronic ankle instability population, ankle surgery history, and animal research |
| I (intervention) | Proprioceptive neuromuscular facilitation(PNF), either alone or in conjunction with other measures | Non-clinical trials and studies without intervention designs |
| C(comparison) | Any alternative treatment includes standard care, placebo, or no treatment |  |
| O (outcomes) | Functional scales、Balance tests、visual analog scale、Muscle strength、Joint Position Sense、Range of Motion | Studies from which data is not extracted or original data are inaccessible |
| S (Setting) | Language of Publication: Articles published in Chinese or English. | Non-Chinese/English documents; non-original research, such as reviews |

Note: Functional scales including the Ankle Joint Functional Assessment Tool Questionnaire (AJFAI) and the Cumberland Ankle Instability Tool (CAIT); Balance tests include the Star Excursion Balance Test (SEBT) in directions: Anterior (ANT), Anterolateral (ALAT), Anteromedial (AMED), Medial (MED), Posterior (POST), Lateral (LAT), Posterolateral (PLAT), and Posteromedial (PMED), as well as the Y Balance Test (YBT); Visual Analog Scale for Pain (VAS); Muscle strength assessments covering relative peak torque during Plantar flexion, Dorsiflexion, Inversion, and Eversion; Inversion Joint Position Sense; and Ankle Dorsiflexion Range of Motion (DFROM).

**2. Provide more specific details on the measurement of outcomes, particularly muscle strength.**

**Thank you for this suggestion. Revisions have been made to the manuscript.**

3.4 Effect of PNF on Muscle Strength

Muscle strength was assessed using both an isokinetic dynamometer and an isometric handheld dynamometer. The former was used to measure the relative peak torque of the muscles [40], whereas the latter evaluated the maximum peak force [14]. Higher values of relative peak torque and maximum peak force indicate greater muscle strength. Since the units of measurement for the two methods differ, the standardized mean difference (SMD) was chosen for data synthesis.

**3. Enhance the discussion by comparing findings with existing literature and providing more specific suggestions for future research.**

**Thank you for this suggestion. Revisions have been made to the manuscript.**

**In the second, third, fourth, and fifth paragraphs of the discussion, the comparison between the research results and the existing literature is added.**

Second paragraph: The results suggest that PNF techniques can improve lower limb postural control, aligning with previous findings [23, 38]

Third paragraph: Compared to other interventions, PNF training also involves additional sensory stimuli (tactile, visual, or verbal) to better facilitate neuromuscular responses and enhance the subject's neural control of the muscles[58].

Fourth paragraph: PNF can alleviate pain, help patients avoid discomfort during movement and daily activities, improve joint function and quality of life, and reduce the risk of secondary injuries.

Fifth paragraph: A meta-analysis conducted by AKASAKI H et al. [64] on the effects of PNF on joint position sense showed that PNF did not provide significant advantages, which is consistent with the results of this study.

**In the article's conclusion, suggestions provided by future research are added.**

In clinical treatment, PNF training is recommended for improving pain, balance ability, and muscle strength in patients with CAI. However, additional therapeutic approaches should be incorporated if patients with CAI experience significant mobility limitations. For future research directions, we propose the following: (1) To optimize research protocols and experimental designs by using PNF as the sole variable in the experimental group, and including other interventions, either without PNF or with sham PNF, in the control group to determine the combined effects of PNF and other interventions; (2) To increase the sample size and conduct medium- and long-term follow-ups on CAI patients treated with PNF to assess whether the improvements are sustained over time; (3) To further conduct high-quality randomized clinical trials in clinical settings to provide more evidence-based support for clinical application.

**4. Address minor grammatical errors and improve formatting consistency**

**Thank you for this suggestion. Revisions have been made to the manuscript.**

The manuscript has been checked and revised.
